# Potential utility of cone‐beam CT‐guided adaptive radiotherapy under end‐exhalation breath‐hold conditions for pancreatic cancer

**DOI:** 10.1002/acm2.13827

**Published:** 2022-10-31

**Authors:** Ayaka Ogawa, Mitsuhiro Nakamura, Hiraku Iramina, Michio Yoshimura, Takashi Mizowaki

**Affiliations:** ^1^ Department of Radiation Oncology and Image‐Applied Therapy Graduate School of Medicine Kyoto University Kyoto Japan; ^2^ Division of Medical Physics Department of Information Technology and Medical Engineering Human Health Sciences Graduate School of Medicine Kyoto University Kyoto Japan

**Keywords:** CBCT‐guided adaptive radiotherapy, dosimetric analysis, independent calculation‐based QA, pancreatic cancer

## Abstract

**Purpose:**

The purpose of this study was to demonstrate the potential utility of cone‐beam computed tomography (CBCT)‐guided online adaptive radiotherapy (ART) under end‐exhalation breath‐hold (EE‐BH) conditions for pancreatic cancer (PC).

**Methods:**

Eleven PC patients who underwent 15‐fraction volumetric‐modulated arc therapy under EE‐BH conditions were included. Planning CT images and daily 165 CBCT images were imported into a dedicated treatment planning system. The prescription dose was set to 48 Gy in 15 fractions. The reference plan was automatically generated along with predefined clinical goals. After segmentation was completed on CBCT images, two different plans were generated: One was an adapted (ADP) plan in which re‐optimization was performed on the anatomy of the day, and the other was a scheduled (SCH) plan, which was the same as the reference plan. The dose distributions calculated using the synthetic CT created from both planning CT and CBCT were compared between the two plans. Independent calculation‐based quality assurance was also performed for the ADP plans, with a gamma passing rate of 3%/3 mm.

**Results:**

All clinical goals were successfully achieved during the reference plan generation. Of the 165 sessions, gross tumor volume *D*_98%_ and clinical target volume *D*_98%_ were higher in 100 (60.1%) and 122 (74.0%) ADP fractions. In each fraction, the *V*_3 Gy_ < 1 cm^3^ of the stomach and duodenum was violated in 47 (28.5%) and 48 (29.1%), respectively, of the SCH fractions, whereas no violations were observed in the ADP fractions. There were statistically significant differences in the dose–volume indices between the SCH and ADP fractions (*p* < 0.05). The gamma passing rates were above 95% in all ADP fractions.

**Conclusions:**

The CBCT‐guided online ART under EE‐BH conditions successfully reduced the dose to the stomach and duodenum while maintaining target coverage.

## 1 INTRODUCTION

Pancreatic cancer (PC) is the fourth leading cause of death in the United States, and morbidity rates have been increasing in recent years.^1^ Most cases are inoperable at the time of initial diagnosis, and multidrug chemotherapy has a prolonged prognosis^2^; however, the 5‐year survival rate is still low at 10%.^1^ Because of the poor prognosis, PC needs to be treated with multimodality therapies, such as surgery, radiation therapy, and chemotherapy. From this point of view, radiation therapy, which can be used in combination with chemotherapy, is increasingly important.

Escalating radiation dose may contribute to the improvement of treatment outcomes^3^, ^4^, ^5^; however, the pancreas is located adjacent to the stomach or duodenum that are sensitive to radiation, and these structures move with respiration,^6^, ^7^ making it difficult to deliver high doses to the lesions even with volumetric‐modulated arc therapy (VMAT). Several studies have been published on the relationship between gastrointestinal (GI) adverse events and dose–volume constraints,^8^, ^9^, ^10^ and the initial treatment plan is typically generated with reference to the published dose–volume constraints. However, the stomach and duodenum have respiratory motion and interfractional physiologic deformation, leading to large dose deviations in both organs and target from the planned dose.^11^, ^12^ From a recent phase I dose‐escalation trial with stereotactic body radiation therapy (SBRT) for PC, it was reported that radiation‐induced severe GI adverse events were observed at a nontrivial rate.^13^ Although offline adaptive radiotherapy (ART) with respiratory motion management technique is one of the approaches to manage respiratory motion and organ deformation, interfractional dosimetric variations are not always predictable even after replanning.

In recent years, the use of online ART, which allows us to immediately generate a new plan reflecting the daily organ deformation, has allowed the delivery of a high dose to the target safely while sparing doses to organs at risk (OARs). There are three types of online ART techniques: magnetic resonance (MR)‐guided,^14^, ^15^, ^16^, ^17^, ^18^, ^19^ computed tomography (CT)‐on‐rails‐guided,^20^, ^21^ and cone‐beam CT (CBCT)‐guided approaches.^22^, ^23^, ^24^, ^25^, ^26^ It is well known that MR images provide superior soft‐tissue contrast and delineation precision compared to CBCT. With the advantages of these characteristics, MR‐guided ART has been clinically applied to PC and has demonstrated its dosimetric advantages.^17^, ^18^, ^19^ ART with CT‐on‐rails has the advantage of daily treatment planning with image quality comparable to that of planning CT; however, there is a positional error associated with couch rotation.^27^ More recently, the image quality of CBCT has been greatly improved, even in the upper GI region owing to high‐speed gantry rotation, enhanced noise‐canceling grid, and advanced image reconstruction algorithms.^28^, ^29^, ^30^ Several studies have demonstrated the clinical efficacy of CBCT‐guided ART in patients with prostate, bladder, cervical, and rectal cancer.^22^, ^23^, ^24^, ^25^, ^26^ Li et al. used in‐house‐developed graphics processing unit‐based replanning system and found the dosimetric utility of daily adaptive replanning with SBRT for PC^31^; however, they did not employ respiratory motion management techniques, and their re‐optimization protocol was based on equal priority, making it difficult to adjust doses, especially to the serial organs. In addition, they did not conduct patient‐specific quality assurance (QA).

In this study, we demonstrated the potential utility of CBCT‐guided online ART under end‐exhalation breath‐hold (EE‐BH) conditions for PC. We created a daily treatment plan on synthetic CT generated from the initial planning CT and daily CBCT in accordance with clinical intent and evaluated the differences in dose distributions to the target and OARs with and without daily adaptation for each session in patients with PC previously treated with 15‐fractionation VMAT under EE‐BH conditions at our institution. In addition to dosimetric comparison, independent calculation‐based QA was performed for daily adapted plans.

## 2 METHODS

### 2.1 Patients

Institutional ethical approval was obtained before the study (approval number: R2762). Twelve consecutive patients with PC who underwent 15‐fraction VMAT under EE‐BH conditions with TrueBeam STx (Varian Medical Systems, Palo Alto, CA, USA) between November 2019 and July 2020 at our institution were enrolled. Of these, one patient (patient 7) was excluded from this study because daily CBCT, which was acquired with a lower mAs value for the patient's body shape, was not available at the segmentation stage (described below) due to extremely poor image quality. Consequently, 11 patients with PC were analyzed in this study (Table 1). All patients had histologically proven pancreatic adenocarcinoma and were diagnosed with borderline resectable or locally advanced unresectable PC on the tumor board at our institution. Oral intake was stopped except for drugs and water for at least 3 h before CT or CBCT acquisition.

**TABLE 1:** Patients’ characteristics

Pt. #	Age (year)	Sex	Primary site	Stage	TNM (UICC 8th)	GTV size (cm^3^)	CTV size (cm^3^)	PTV size (cm^3^)	Overlap ratio (%)
1	72	M	Body	BR	T4N0M0	12.3	94.9	173.1	10.7
2	59	F	Body	BR	T4N1M0	111.6	217.5	348.1	18.5
3	71	M	Head	BR	T4N0M0	38.1	144.3	249.2	15.2
4	79	M	Head	UR	T4N0M0	13.7	101.2	198.9	12.7
5	57	M	Body	UR	T4N0M0	87.3	203.0	330.7	8.9
6	71	F	Head	BR	T2N0M0	31.3	119.1	119.1	16.7
8	78	M	Head	BR	T2N0M0	5.3	104.2	190.9	6.9
9	78	M	Body	BR	T4N0M0	17.9	103.2	187.3	11.5
10	69	M	Body	BR	T2N0M0	18.2	133.7	236.2	15.2
11	64	F	Head	BR	T4N0M0	28.1	91.6	184.8	15.6
12	62	F	Head	UR	T4N0M0	89.8	184.0	297.7	11.6

*Note*: Patient 7 was excluded from this study because daily CBCT, which was performed with a lower mAs value for the patient's body shape, was not available at the segmentation stage due to extremely poor image quality. Overlap refers to the area where the PTV and PRV overlap. The overlap ratio was calculated as the overlap volume to the PTV volume.

Abbreviations: BR, borderline resectable; CBCT, cone‐beam computed tomography; CTV, clinical target volume; GTV, gross tumor volume; PRV, planning organs at risk volume; PTV, planning target volume; UR, unresectable.

### 2.2 Initial planning CT acquisition, contouring, and daily CBCT acquisition

All patients were immobilized in a headfirst supine position with both upper limbs elevated using an individualized vacuum pillow (Body Fix; Elekta, Stockholm, Sweden). Non‐contrast and transvenous contrast CT were then acquired under EE‐BH conditions with a real‐time position management system (RPM; Varian Medical Systems). The operator instructed the patient to hold their breath while monitoring the patient's respiratory signal with the RPM system. Patients held their breath according to the operator's instruction only without visual feedback. This was done in accordance with a previous study where further details can be found.^32^

The gross tumor volume (GTV) included the primary tumor and metastatic lymph nodes. The clinical target volume (CTV) was defined as the GTV plus a 5 mm margin and the potential para‐aortic lymph node and neuroplexus involvement between the celiac axis and the superior mesenteric artery. A planning target volume (PTV) margin of 5 mm was isotropically added to the CTV. The surrounding OARs, such as the stomach and duodenum, were also contoured. Planning organs at risk volume (PRV) margins of 3‐ and 5 mm were added to the duodenum and stomach, respectively, based on previous data.^33^ These structures were defined by a board‐certified radiation oncologist.

On the day of treatment, CBCT was acquired just before beam delivery under EE‐BH conditions with the RPM system. One gantry rotation was divided into three to four segments to acquire CBCT images, depending on the patient's BH ability. The CBCT field‐of‐view (FOV) was 26.3 cm, and the slice thickness was 2 mm. The images were reconstructed using an iterative reconstruction algorithm with a medium noise suppression level.^34^

### 2.3 Workflow of CBCT‐based online adaptive radiotherapy

**FIGURE 1:**
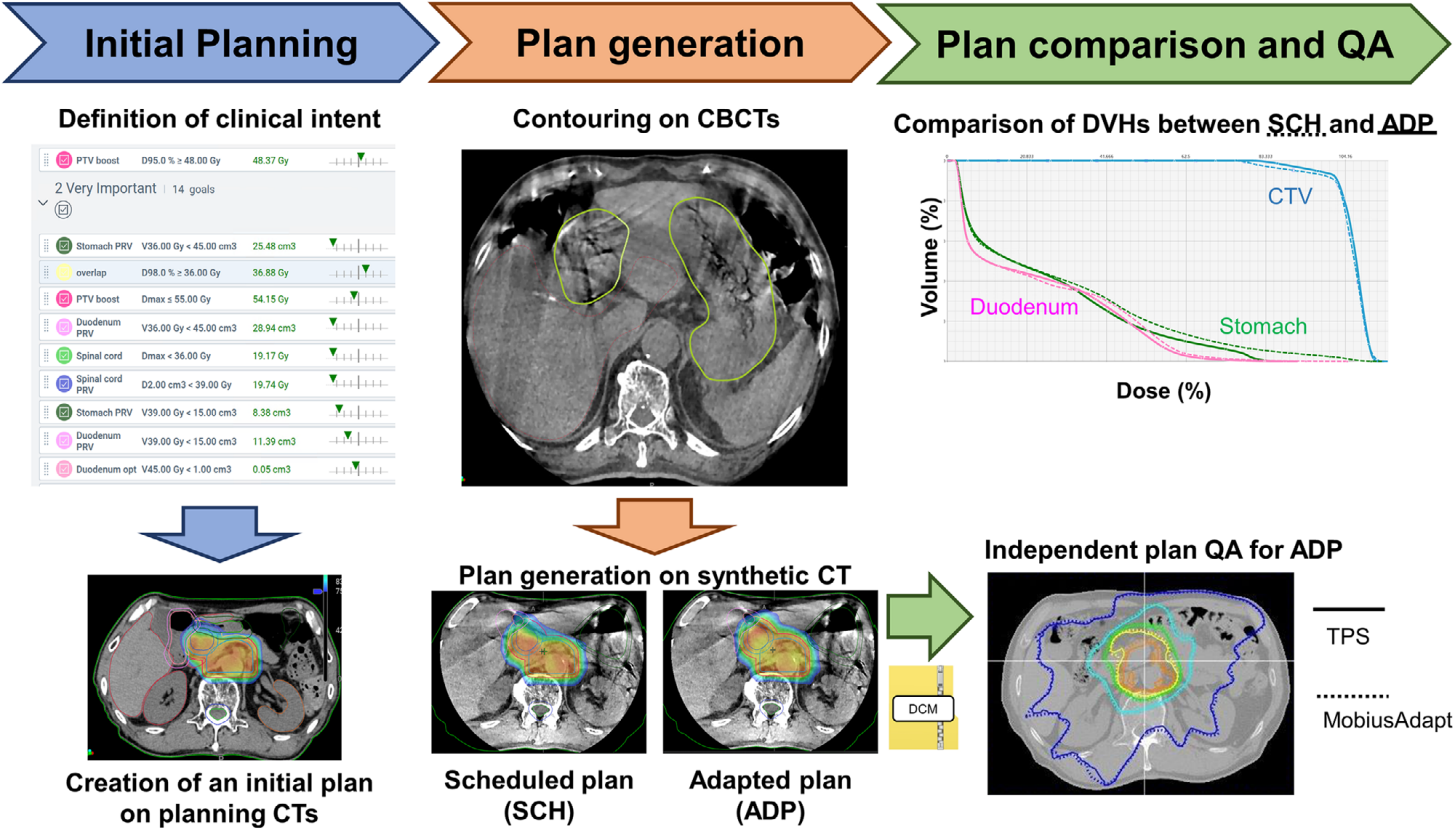
Overview of the present study

The overall workflow of this study is shown in Figure 1, which consisted of initial planning, daily plan generation, and independent calculation‐based QA.

The initial planning CT and structures used for the previous treatment were imported to the Ethos therapy solution emulator version 1.1 (Varian Medical Systems). Reference plans with 3‐arc VMAT were generated according to an institutional treatment protocol for locally advanced PC. Dose distributions were calculated using Acuros XB with a 2.5 × 2.5 × 2.5 mm^3^ grid resolution. The prescription dose was 48 Gy in 15 fractions with a dose covering 95% of the target (*D*_95%_) to the PTV‐PRV, a volume that subtracted PRVs from the PTV. A PTV‐PRV of *D*_95%_ ≥ 48 Gy and PTV *D*_98%_ ≥ 36 Gy were preferable; however, if this was difficult to achieve within the constraints of the GI tract, the PTV‐PRV and PTV were reduced to *D*_95%_ ≥ 45.6 Gy and *D*_98%_ ≥ 34.2 Gy, respectively. The dose–volume constraints for OARs were also the same as in a previous institutional trial.^35^ All clinical goals, the dose–volume constraints and the priority are shown in Table 2.

**TABLE 2:** Clinical goals and priority used for the reference plan and adaptive plan

Priority	Importance	Structure	Goals	Acceptable variation
1	Most important	PTV‐PRV^a^	*D*_95%_ ≥ 48 Gy (*D*_95%_ ≥ 45.6 Gy)^b^	
2	Very important	Stomach PRV^a^	*V*_36 Gy_ < 45 cm^3^	
2	Very important	Overlap	*D*_98%_ ≥ 36 Gy (*D*_98%_ ≥ 34.2 Gy)^b^	
2	Very important	PTV‐PRV	*D*_max_ < 55 Gy	*D*_max_ < 56 Gy
2	Very important	Duodenum PRV^a^	*V*_36 Gy_ < 45 cm^3^	
2	Very important	Spinal cord^a^	*D*_max_ < 36 Gy	
2	Very important	Spinal cord PRV^a^	$D_{2cm^{3}}$ < 39 Gy	
2	Very important	Stomach PRV^a^	*V*_39 Gy_ < 15 cm^3^	*V*_39 Gy_ < 30 cm^3^
2	Very important	Duodenum PRV^a^	*V*_39 Gy_ < 15 cm^3^	*V*_39 Gy_ < 30 cm^3^
2	Very important	Duodenum^a^	*V*_45 Gy_ < 1 cm^3^	
2	Very important	Duodenum^a^	*V*_42 Gy_ < 5 cm^3^	
2	Very important	Duodenum^a^	*V*_39 Gy_ < 25 cm^3^	
2	Very important	Stomach^a^	*V*_45 Gy_ < 1 cm^3^	
2	Very important	Stomach^a^	*V*_42 Gy_ < 5 cm^3^	
2	Very important	Stomach^a^	*V*_39 Gy_ < 25 cm^3^	
3	Important	Lt kidney^a^	*V*_20 Gy_ < 30%	
3	Important	Rt kidney^a^	*V*_20 Gy_ < 30%	
3	Important	Liver^a^	*D*_mean_ < 30 Gy	
3	Important	Overlap	*D*_5%_ ≤ 48 Gy	*D*_5%_ ≤ 49 Gy
3	Important	PTV‐PRV	*D*_50%_ ≥ 51 Gy	*D*_50%_ ≥ 50 Gy

Abbreviations: *D*_max_, maximum dose; *D*_mean_, mean dose; *D_xx_*_%_, dose covering *xx*%; Lt, left; PRV, planning organ at risk volume; PTV, planning target volume; Rt, right; *V_xx_* _Gy_, the volume received by *xx* Gy.

^a^Dose–volume constraints are the same in our facility for locally advanced pancreatic cancer.

**^b^** As one patient (patient 2) did not meet the clinical goals due to a large overlapping volume between PTV and PRVs, the case was allowed a PTV‐PRV of *D*_95%_ ≥ 45.6 Gy (95% of 48 Gy) and PTV of *D*_98%_ ≥ 34.2 Gy (95% of 36 Gy).

Daily CBCT images for each patient were also imported into the Ethos emulator in sequence. The following adaptive session was performed for each CBCT: First, the predefined OARs (duodenum, stomach, and liver) were automatically segmented using full image deep convolutional neural networks with proprietary architectures that share many similarities with U‐Net and DenseNet implemented on the Ethos system.^34^ In this study, the duodenum, stomach, and liver were automatically segmented. The contours for the other OARs (kidneys and spinal cord) were then automatically propagated from the initial planning CT using a modified basis spline (B‐spline) deformable image registration (DIR) algorithm, followed by a simple post‐processing and smoothing of the results.^36^ The small and large intestines were not contoured on the CBCT images in this study. In contrast, the targets were rigidly propagated from the initial planning CT via rigid registration. These structures should be reviewed and manually modified until clinically acceptable levels were reached by a board‐certified radiation oncologist in reference to the initial planning CT when needed.

Thereafter, the adapted (ADP) plan was generated under the same clinical goals as the reference plan, with daily approved contours. Optimization and dose distribution were conducted on the daily synthetic CT. The synthetic CT was generated by deforming the initial planning CT using the B‐spline DIR algorithm.^36^ To compensate for the limited FOV as well as the motion artifacts on a CBCT, each CBCT voxel Hounsfield unit value was replaced with those on the planning CT. Furthermore, the planning CT voxel information was propagated via a rigid registration to the areas outside the FOV of the CBCT to compensate for the limited scan length. In order to increase registration quality for CT‐to‐CBCT DIR, only those voxels that lied inside the CBCT reconstruction radius were used in the cost function. This avoided registration artifacts in cases where the reconstruction radius truncates the body. In parallel with the ADP plan generation, the dose distribution was calculated on the daily synthetic CT using the initial plan information, which was termed the scheduled (SCH) plan. After calculating the dose distributions, the absence of inappropriate hot spots outside the target was confirmed visually.

### 2.4 Dosimetric analysis

All plans were exported to the Eclipse version 16.1 (Varian Medical Systems), and dose–volumetric indices (DVIs) in the stomach, duodenum, GTV, and CTV were examined for the ART and SCH fractions.

These DVIs were analyzed statistically between the ADP and SCH fractions using the Wilcoxon rank sum test. The significance level was set at 5%. All statistical analyses were conducted using EZR (Saitama Medical Center, Jichi Medical University, Japan).

### 2.5 Independent calculation‐based QA

The DICOM dataset (CT images, RT structures, RT plan, and RT dose) was exported to an independent dose calculation software, MobiusAdapt (Mobius Medical Systems, LLC, Houston, TX, USA). Currently, MobiusAdapt is the only software to conduct independent calculation‐based QA for the Ethos system. A collapsed cone convolution/superposition algorithm was implemented as the dose calculation algorithm. MobiusAdapt calculated dose distribution based on the RT plan and then compared it with the dose distribution stored in RT dose. The imported RT dose was used as reference. In this study, the global gamma passing rates (3%/2 and 3%/3 mm) with 10% dose threshold criteria were evaluated.^22^, ^26^, ^37^, ^38^

To investigate the change in the gamma passing rate, Pearson's correlation between the gamma passing rate and monitor units (MUs) difference between the reference plan and ADP fractions or overlap ratio of PRVs to PTV was analyzed.

### 2.6 Time evaluation

The time required to complete segmentation, optimization, and dose calculation was measured with a stopwatch or extracted from the system log.

## 3 RESULTS

### 3.1 Initial plan generation

All clinical goals listed in Table 2 were successfully achieved at the stage of initial plan generation, except for one patient (patient 2) who had a large overlap volume between PTV and PRVs. The clinical goals shown in parentheses for the target volumes were applied in this case (Table 2).

### 3.2 Comparison of the ADP plan with the SCH plan

**FIGURE 2:**
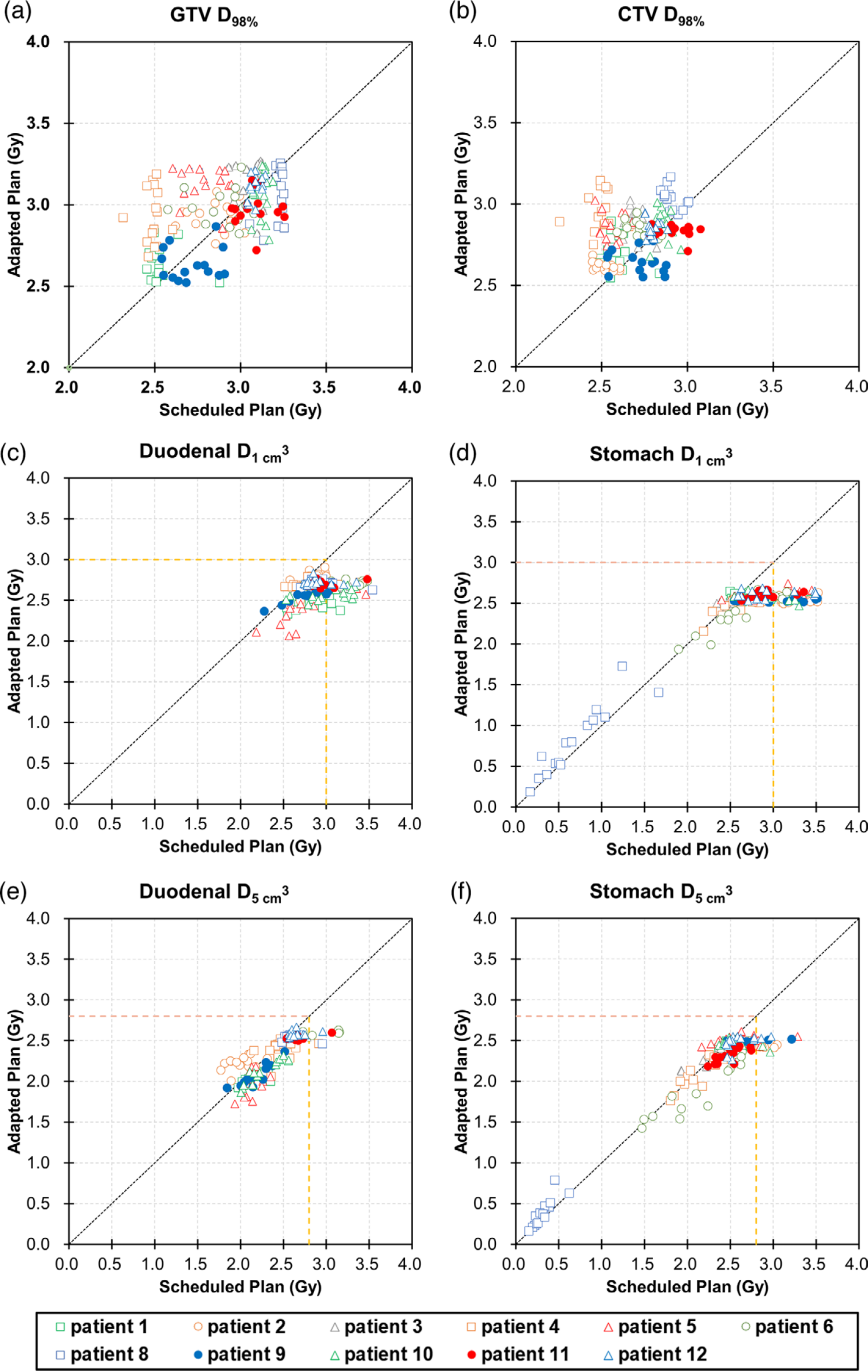
Scatter plots of the dose–volume indices of (a) gross tumor volume (GTV) *D*_98%_, (b) clinical target volume (CTV) *D*_98%_, (c) duodenal $D_{1cm^{3}}$, (d) stomach $D_{1cm^{3}}$, (e) duodenal $D_{5cm^{3}}$, and (f) stomach $D_{5cm^{3}}$ in the scheduled and adapted fractions. If the plots are below the dotted line, it means that the value in the scheduled plan is higher than the value in the adapted plan. The color‐filled circles mean that CTV *D*_98%_ was higher in the scheduled plan than in the adapted plan in more than half of the 15 fractions. The yellow dashed lines in (c)–(f) show the dose–volume constraints used in this study.

**FIGURE 3:**
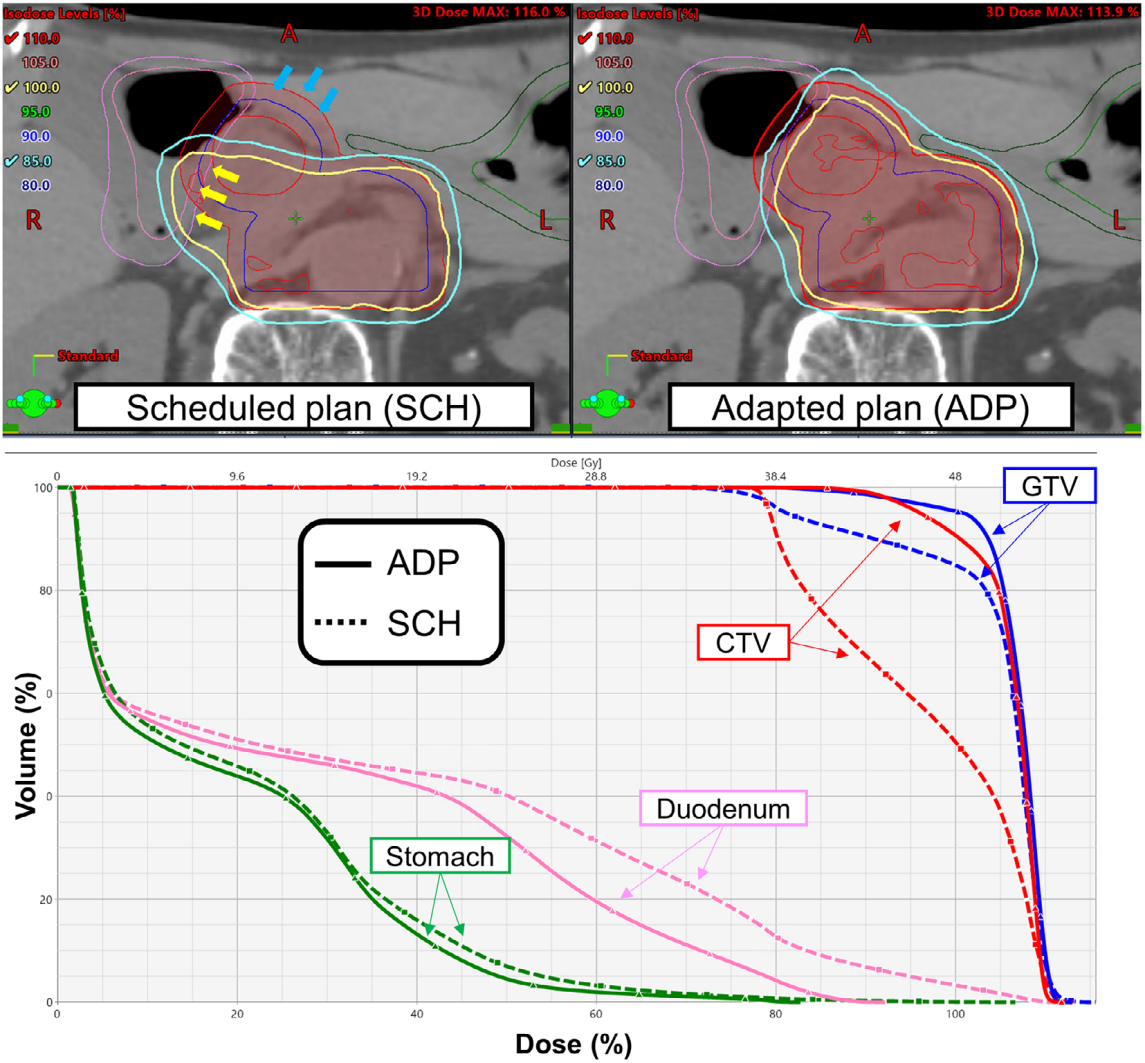
Representative (upper) dose distribution and (lower) dose–volume histograms for which the adaptive plan was effective. In the scheduled plan, the isodose lines indicated by yellow arrows (greater than 100% dose) largely cover the duodenum, and clinical target volume (CTV) coverage was insufficient as shown by the blue arrows; however, these two drawbacks were resolved in the adapted plan.

A total of 165 sessions (11 patients × 15 CBCT images) were analyzed. Figure 2 shows the scatter plots of the DVIs of the GTV, CTV, duodenum, and stomach for the ADP and SCH fractions. Of 165 sessions, GTV *D*_98%_ and CTV *D*_98%_ were higher in 100 (60.1%) and 122 (74.0%) ADP fractions, respectively. The median doses of GTV *D*_98%_ and CTV *D*_98%_ were 2.96 and 2.72 Gy in SCH fractions and 2.98 and 2.84 Gy in ADP fractions, respectively. Meanwhile, the *V*_3 Gy_ < 1 cm^3^ of the stomach and duodenum was violated in 47 (28.5%) and 48 (29.1%) SCH fractions, respectively, whereas no violations were observed in the ADP fractions (Figure 2c,f). The median doses of the stomach and duodenum $D_{1cm^{3}}$ were 2.75 and 2.88 Gy in SCH, and 2.54 and 2.67 Gy in ADP fractions, respectively. There were statistically significant differences in the DVIs between the SCH and ADP fractions (*p* < 0.05). Figure 3 shows a typical example of dose distribution and dose–volume histograms. In the SCH fractions, the 100% isodose line (a surrogate for 45 Gy in 15 fractions) largely covered the duodenum, and dose coverages to the GTV and CTV were insufficient; however, these two drawbacks were resolved in the ADP plan.

The overlap ratio of the stomach or duodenum PRV to PTV at the initial planning had a potential predictor for the number of violations of *V*_42 Gy_ > 5 cm^3^ in the SCH fractions (Figure S1). The correlation coefficients were 0.85 and 0.69 for the stomach *V*_42 Gy_ and duodenum *V*_42 Gy_, respectively. These results indicated that the violations did not occur randomly but tended to occur in specific patients.

### 3.3 Independent calculation‐based QA

**FIGURE 4:**
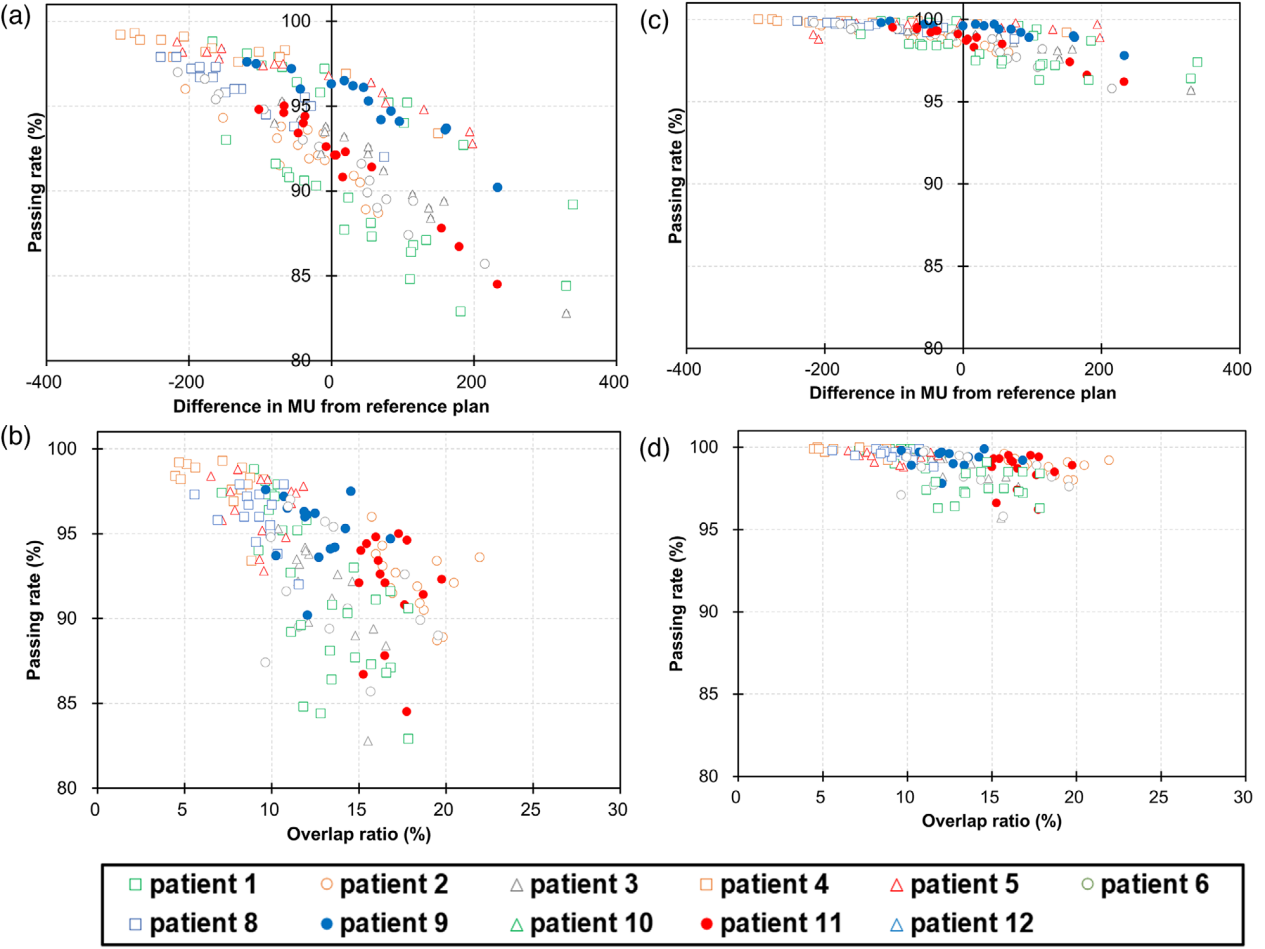
The gamma passing rate as a function of (a and c) the difference in monitor units (MUs) from the reference plan and (b and d) overlap ratio of the planning organs at risk volumes (PRVs) to planning target volume (PTV). (a and b) A gamma passing rate of 3%/2 mm, and (c and d) a gamma passing rate of 3%/3 mm

The mean ± standard deviation of the gamma passing rate was 93.5% ± 3.7% (range 82.8%–99.3%) and 98.9% ± 0.9% (range 95.7%–100.0%) with gamma criteria of 3%/2 and 3%/3 mm, respectively. Figure 4 shows the relationship between the difference in the MU from the reference plan or the overlap ratio and gamma passing rate, with the gamma criteria of 3%/2 and 3%/3 mm. The gamma passing rate decreased with a larger MU, and the correlation coefficient was −0.75 and −0.70, with the gamma criteria of 3%/2 and 3%/3 mm, respectively (Figure 4a,c). In addition, the correlation coefficient between the overlap ratio and gamma passing rate was −0.66 and −0.54, with the gamma criteria of 3%/2 and 3%/3 mm, respectively (Figure 4b,d). Of 165 ADP fractions, 29 (17.6%) had a gamma passing rate below 90% with the gamma criterion of 3%/2 mm. Of these 29 ADP fractions, 19 (65.5%) violated the dose–volume constraints for OARs if the SCH fraction was selected.

### 3.4 Time evaluation

The means ± standard deviations of the time to complete segmentation, optimization, and dose calculation were 537.6 ± 107.1 s (range 378.0–723.0 s), 305.4 ± 20.6 s (range 239.6–359.7 s), and 28.8 ± 1.0 s (range 26.9–31.4 s), respectively.

## 4 DISCUSSIONS

This study aimed to demonstrate the clinical feasibility of CBCT‐based online ART for PC. We showed that 28.5% and 29.1% of SCH fractions violated the dose–volume constraints for the stomach and duodenum, respectively, whereas no violations for these organs were observed without reducing the target coverage of ADP fractions, and 60.1% and 74.0% of the ADP fractions had higher GTV *D*_98%_ and CTV *D*_98%_ than for the SCH fractions. The absence of a violation ensures that the Ethos system successfully optimized the ADP plan regardless of patient anatomy.

It is difficult to estimate the daily dose to the stomach and duodenum during initial planning because interfractional variations are unpredictable. Niedzielski et al. recently reported large dosimetric uncertainties resulting from interfractional variations in patients with PC; in four out of 11 patients, duodenal $D_{1cm^{3}}$ received a higher dose of 1 Gy per fraction than the planned dose.^12^ The impact is especially pronounced in SBRT. Courtney et al. observed grades 4–5 late toxicities in a phase I dose‐escalation trial of SBRT for PC without online ART.^13^ However, recent stereotactic MR‐guided online ART studies reported that an incident rate of grade 3 GI toxicity was acceptable after high‐dose SBRT (50 Gy in five fractions).^17^, ^18^ From these findings, the observation of dose–volume constraints of every single day would be essential, which can be realized with CBCT‐guided online ART.

Although there were differences in prescription doses, PRV margin sizes, and chemotherapy, previous studies have shown that the frequency of GI toxicity increases when certain dose–volume constraints are not achieved in the initial plan^8^, ^9^, ^10^ From the present study, it is possible to understand the daily dose distribution. Although the visualization of the dose distribution for each session is a major progress, the accumulated dose of OAR should be a better indicator of adverse events. The DIR is one of the approaches to obtain the accumulated dose; however, its accuracy is low, especially in abdominal and pelvic regions.^39^, ^40^ Ziegler et al. accumulated the delivered dose for pancreatic patients based on CBCTs using DIR; however, they pointed out that there were still large residual registration errors, such as huge movement of air pocket.^39^ Thus, a few CBCT datasets with severe artifacts were unavailable for dose accumulation in their study. Swamidas et al. pointed out that the current DIR algorithms are not yet robust.^40^ Developments of reliable accumulated dose and clinical results are expected in the future.

To reduce the intrafractional error with online adaptive therapy, respiratory motion management should obviously be used, as the pancreas and OARs have been reported to move more than 10 mm along with respiration.^6^, ^7^, ^41^ In the current clinical practice, RPM (Varian Medical Systems) cannot be used with the current Ethos version. If venders implement their own respiratory motion management devices in the near future or facilities use third‐party ones appropriately, such as AlignRT (Vision RT, London, UK)^42^ and SpiroDynr'X system (SDX) (DYN'R, Aix‐en‐Provence, France),^43^ our clinical workflow can be achieved.

In this study, we did not assess the dose to the bowel, considering their clinical impact and the reliability of the contouring. In our clinical intensity‐modulated radiotherapy (IMRT), the PRV for bowels was not generated because it was not always in the same position due to peristalsis and daily change in the amount of bowel content, and no critical adverse events have been observed so far.^33^, ^44^ Although McGinn et al. reported transverse colon stenosis in a dose‐escalation trial of 15 fractions,^45^ they did not use IMRT. When employing hypofractionation, such as 5 fractions, it is more important to evaluate the bowel dose even if it takes more time to adaptation. In the Ethos platform, the high dose areas are automatically optimized not to extend beyond the target, and the radiation oncologist and medical physicist confirmed visually that the 45 Gy out of the PTV did not cover the bowel.

Patient‐specific QA is required to verify treatment delivery and dose calculation by the treatment planning system. Although patient‐specific QA is typically based on measurements, calculation‐based QA is the standard for online ART because the patient is on the treatment couch during daily plan generation.^38^ In this study, independent calculation‐based QA was conducted for all ADP fractions with the gamma criteria of 3%/2 and 3%/3 mm. With the gamma criterion of 3%/2 mm recommended by AAPM TG218,^37^ 29 (17.6%) and 5 (3.0%) fractions had gamma passing rates below 90% and 85%, respectively. If these criteria are employed in clinical practice, some ADP fractions will not be acceptable. In contrast, the gamma passing rates were above 95% with the gamma criteria of 3%/3 mm, which were used by several investigators,^22^, ^26^ in all ADP fractions. Even with the gamma criteria of 3%/2 and 3%/3 mm, the gamma passing rate exhibited negative correlation with the MU difference between the reference plan and ADP fractions or the overlap ratio, which were supported by the results of Sibolt et al.^46^ As stated in another study by Sibolt et al.,^22^ higher modulation factor (MU/Gy) might contribute to the negative correlation. AAPM TG219 recommends that users develop and use confidence limits from clinical data points. From our findings and the recommendation from AAPM TG219, careful determinations of the tolerance and action level are required before clinical application at each facility.

Online ART workflow itself is time‐sensitive; thus, time evaluation is essential. As expected, the time to complete segmentation was the longest, followed by optimization and dose calculation. As the accuracy of the segmentation determines the quality of the dose distribution, careful confirmation of the contouring is necessary. There is room for reconsideration regarding the time required for optimization. Additional internal review revealed that the time required for optimization was reduced by one third regardless of the number of ports when fixed gantry IMRT is selected. If the dose distribution is equivalent between IMRT and VMAT, IMRT would be the more practical choice.

This study has several limitations. First, the number of OAR violations during the course of treatment has not been shown to lead to severe adverse clinical events. Accurate determination of the clinical threshold is expected, as actual dose and clinical results are accumulated in the future. The second is contouring error. Even though the CBCT image quality has improved, artifacts caused by air pockets are still present, which were observed around stomach, duodenum, and bowels. As stated in the Methods section, OAR contours were visually checked and manually corrected if necessary. Niedzielski et al. concluded that the effect of contouring variability on the OAR $D_{1cm^{3}}$ was negligible by expanding or shrinking structures contoured on CT‐on‐rail images.^12^ In this study, dose–volume constraints for not only OAR $D_{1cm^{3}}$ but $D_{5cm^{3}}$ were fulfilled, which would ensure the accurate delineation of OAR contours. The shape of the GTV is less deformable than the peristaltic intestine; therefore, the same shape at the initial planning CT can be used for daily plan generation. Taking oral contrast agents prior to treatment may be useful for improving image quality.^47^ The third is the intrafraction variation. Intrafraction variations occurred immediately after the CBCT acquisition. One study reported that intramuscular butylscopolamine improved the MR image quality of the pancreas by suppressing GI peristalsis^48^; thus, premedication may reduce intrafractional error.

## 5 CONCLUSIONS

The clinical feasibility of CBCT‐guided online ART for PC was investigated. Dose distributions were compared between ADP and SCH fractions using 15‐fraction VMAT. We have demonstrated that the ADP fractions met the dose–volume constraints in each session, although 28.5% and 29.1% of SCH fractions violated the stomach and duodenum doses, respectively. From the results of the independent calculation‐based QA, more MUs from the initial plan or a larger overlap ratio of PRVs to PTV would cause a decrease in the gamma passing rate with the criteria of 3%/2 mm; however, the decline was mitigated with the criteria of 3%/3 mm. In conclusion, CBCT‐guided online ART successfully reduced the dose to the stomach and duodenum while maintaining target coverage.

## AUTHOR CONTRIBUTIONS

Ayaka Ogawa acquired, processed, and analyzed the data and wrote the manuscript. He also performed the statistical analysis. Mitsuhiro Nakamura and Hiraku Iramina helped to acquire the data, provided input for the study, and helped to write the manuscript. Mitsuhiro Nakamura, Michio Yoshimura, and Takashi Mizowaki conceptualized the project and oversaw all aspects of the work. All the authors reviewed and provided feedback on the manuscript.

## CONFLICT OF INTEREST

Prof. Mizowaki and Dr. Nakamura have a collaborative research agreement with Varian Medical Systems. All other authors have no conflict of interest.

## Supporting information

Supporting Information

## Data Availability

Research data are stored in an institutional repository and will be shared upon request to the corresponding author.
