# Supplementary material for: Potential utility of cone‐beam CT‐guided adaptive radiotherapy under end‐exhalation breath‐hold conditions for pancreatic cancer
Source: J Appl Clin Med Phys. 2022 Oct 31;24(2):e13827. doi: 10.1002/acm2.13827 (PMC9924116; doi:10.1002/acm2.13827)
Supplement: Supplementary file 1 — Supporting Information [file ACM2-24-e13827-s001.docx]

**Supplementary file**

While most of the previous online ART studies were based on 5-fraction SBRT, our study was on 15-fraction VMAT. We have traditionally performed 15-fraction of moderate-hypofractionated VMAT. The merit of this treatment strategy is that we can use chemotherapy concurrently and include a part of the lymph node areas [1,2]. Concurrent chemotherapy can be expected to have a synergistic effect with radiotherapy, and there is no need for chemotherapy interruption in PC, which contributes in suppressing distant metastasis. As for the elective nodal regions, ASTRO guidelines indicate that SBRT does not routinely treat them [3]; however, according to a study on patterns of local failure of SBRT, the areas closer to the celiac trunk, superior mesenteric artery, and retroperitoneal space were categorized into the high-risk patient group of recurrence [4]. With recent few exceptions [5,6], it is not common to include the elective nodal regions in SBRT. Some clinical studies in which the PTV included the elective nodal regions used a 15-fraction regimen, and their treatment outcomes were favorable [1,7]. Thus, the 15-fraction regimen is promising for improving treatment outcomes in patients with large PTV. A large PTV includes parts of OARs; therefore, online ART will be helpful in 15-fraction regimens as well as 5-fraction SBRT.

Currently, online ART still requires substantial resources [8], and achieving online ART is difficult for all cases; therefore, it is also important to predict high-risk patient groups with significant interfractional variations to avoid severe GI toxicity. Bohoudi et al. concluded that patients whose distance between the GTV and OARs was ≤ 3 mm should be categorized into the high-risk group [9]. In this study, for all patients, the GTV was in contact with the stomach or duodenum. The present study showed that the frequency of deviation from dose-volume constraints tended to be patient-dependent, even though the distance between the GTV and OARs was 0 mm. In particular, the number of times V_42 Gy_ was above 5 cm^3^ had a strong correlation with the overlap ratio of PRVs to PTV at initial planning (**Supplementary Figure 1**); thus, patients with a large overlap volume ratio at the initial planning might be categorized into high-risk patient groups requiring online ART.

**References**

1. Goto Y, Nakamura A, Ashida R, et al. Clinical evaluation of intensity-modulated radiotherapy for locally advanced pancreatic cancer. *Radiat Oncol* 2018;13:118.
2. Iwai T, Yoshimura M, Ashida R, et al. Hypofractionated intensity-modulated radiotherapy with concurrent chemotherapy for elderly patients with locally advanced pancreatic carcinoma. *Radiat Oncol* 2020;15:264.
3. Palta M, Godfrey D, Goodman KA, et al. Radiation Therapy for Pancreatic Cancer: Executive Summary of an ASTRO Clinical Practice Guideline. *Pract Radiat Oncol* 2019;9:322-332.
4. Zhu X, Ju X, Cao Y, et al. Patterns of local failure after stereotactic body radiation therapy and sequential chemotherapy as initial treatment for pancreatic cancer: implications of target volume design. *Int J Radiat Oncol Biol Phys* 2019;104:101-110.
5. Chuong MD, Bryant J, Mittauer KE, et al. Ablative 5-fraction stereotactic magnetic resonance-guided radiation therapy with on-table adaptive replanning and elective nodal irradiation for inoperable pancreas cancer. *Pract Radiat Oncol* 2021;11:134-147.
6. Miller JA, Toesca DAS, et al. Pancreatic stereotactic body radiation therapy with or without hypofractionated elective nodal irradiation. *Int J Radiat Oncol Biol Phys* 2021 (in press).
7. Reyngold M, O’Reilly EM, Varghese AM, et al. Association of ablative radiation therapy with survival among patients with inoperable pancreatic cancer. *JAMA Oncol* 2021;7:735-738.
8. Henke L, Kashani R, Robinson C, et al. Phase I trial of stereotactic MR-guided online adaptive radiation therapy (SMART) for the treatment of oligometastatic or unresectable primary malignancies of the abdomen. *Radiother Oncol* 2018;126:519-526.
9. Bohoudi O, Bruynzeel AME, Meijerink MR, et al. Identification of patients with locally advanced pancreatic cancer benefitting from plan adaptation in MR-guided radiation therapy. *Radiother Oncol* 2019;132:16-22.

**
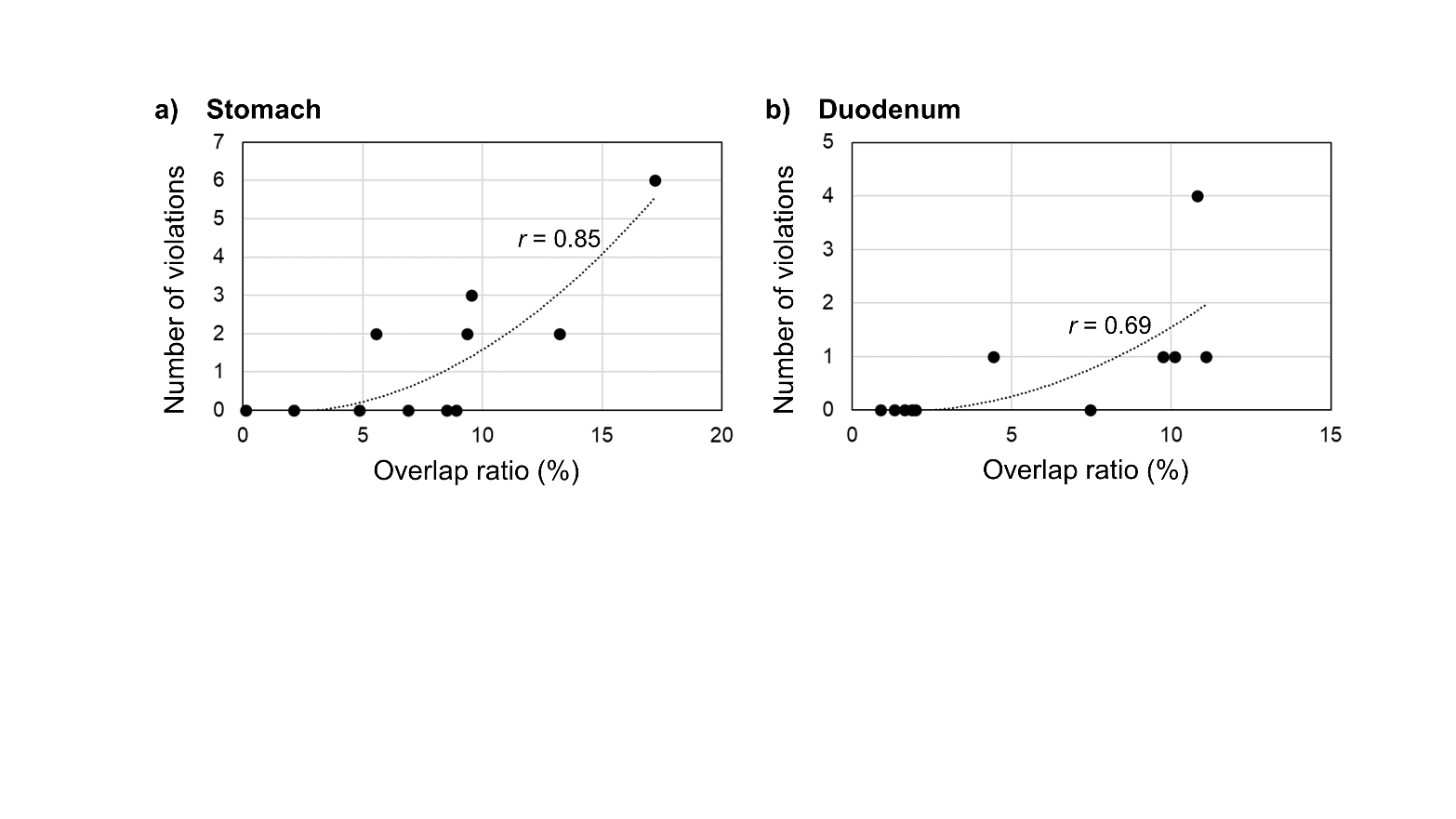
**

**Supplementary Figure 1.** Number of violations as a function of the overlap ratio of the stomach or duodenum PRV to PTV at the initial planning. The overlap ratio had a potential predictor for the number of violations of V_42 Gy_ > 5 cm^3^ in the SCH plans. The correlation coefficients were 0.85 and 0.69 for the stomach V_42 Gy_ and duodenum V_42 Gy_, respectively.
